# Mediterranean dietary pattern and non-alcoholic fatty liver diseases: a case-control study

**DOI:** 10.1017/jns.2021.43

**Published:** 2021-07-26

**Authors:** Mohammad-Reza Entezari, Nasir Talenezhad, Farhang Mirzavandi, Shahab Rahimpour, Hassan Mozaffari-Khosravi, Hossein Fallahzadeh, Mahdieh Hosseinzadeh

**Affiliations:** 1 Shahid Sadoughi University of Medical Sciences and Health Services, Yazd, Iran; 2 Department of Biostatistics and Epidemiology, Research Center of Prevention and Epidemiology of Non-Communicable Disease, Faculty of Health, Shahid Sadoughi University of Medical Sciences, Yazd, Iran; 3 Nutrition and Food Security Research Center, Shahid Sadoughi University of Medical Sciences, Yazd, Iran; 4 Department of Nutrition, School of Public Health, Shahid Sadoughi University of Medical Sciences, Yazd, Iran

**Keywords:** Mediterranean diet, Non-alcoholic fatty liver disease, Dietary pattern, ALT, alanine aminotransferase, AST, aspartate aminotransferase, HC, hip circumference, MED, Mediterranean, MUFA/SAFA, monounsaturated fatty acid/polyunsaturated fatty acid, NAFLD, non-alcoholic fatty liver diseases, TC, total cholesterol, TAG, triacylglycerols, WC, waist circumference

## Abstract

The Mediterranean (MED) diet was associated with a reduced risk of chronic disease, but the epidemiological studies reported inconsistent findings related to the MED diet and non-alcoholic fatty liver disease (NAFLD) risk. This age and the gender-matched case-control study were conducted among 247 adult patients. The MED diet score was obtained based on the Trichopoulou model. Multivariate logistic regression was used to examine the association between the MED diet and NAFLD risk. NAFLD prevalence in people with low, moderate and high adherence to the MED diet was 33, 13⋅1 and 4⋅6 %, respectively. The increasing intake of the MED diet was significantly related to the increment intake of nuts and fruits, vegetables, monounsaturated fatty acid/polyunsaturated fatty acid ratio, legumes, cereals and fish. However, total energy consumption, low-fat dairy and meats intake were reduced (*P* for all < 0⋅05). Following control for age, the person in the highest of the MED diet tertile compared with the lowest, the odds of NAFLD decreased (OR: 0⋅40, 95 % CI: 0⋅17–0⋅95). This relation became a little stronger after further adjusting for sex, diabetes, physical activity and supplement intake (OR: 0⋅36, 95 % CI: 0⋅15–0⋅89). However, this association disappeared after adjusting for body mass index, waist and hip circumference (OR: 0⋅70, 95 % CI: 0⋅25–1⋅97). High adherence to the MED diet was associated with a 64 % reduction in NAFLD odds before some anthropometric variable adjustments. However, further prospective studies are required, particularly in BMI-stratified models.

## Introduction

Non-alcoholic fatty liver disease (NAFLD) is the most common cause of chronic liver disease worldwide^(1)^. NAFLD includes a wide range of liver damage, from simple steatosis to cirrhosis^(2)^. NAFLD prevalence is estimated in adults worldwide, and it is evaluated to be about 20–25, 5–18 and 21⋅5–31⋅5 % in Asian countries and Iranian populations, respectively^(3–5)^. Obesity, diabetes, hyperlipidaemia, inadequate physical activity and an unhealthy diet are the most critical risk factors for NAFLD^(6–9).^

Over the past decades, nutrition has been considered the dominant factor in preventing and treating non-communicable diseases, such as depression^(10)^ and NAFLD. Many studies demonstrated that deficiency in some micro- or macronutrients and high intake of some food groups could be related to NAFLD^(10–12)^. In recent years, epidemiological studies have shown that dietary patterns can better understand the correlation between dietary intake and NAFLD. The Mediterranean dietary score (MED diet) was proposed for the first time in 1995^(13)^. This dietary pattern was designed based on usual dietary intake and related to the risk of cardiovascular risk factors in MED countries. The MED diet includes a lot of beneficial fatty acids, fibre and micronutrients^(14)^. Some epidemiological studies showed that NAFLD risk was reduced with increased adherence to the MED diet^(15–20)^, even though others could not find such a relationship^(15–17)^. To the best of our knowledge, a few studies investigated the MED diet in developing countries, especially middle east countries where dietary patterns are different from other parts of the world. So, this case-control study aimed to examine the association between adherence to the MED diet and NAFLD in Iranian adults.

## Material and methods

In this case-control study, we examined the 247 adult participants (108 men and 139 women) with an age range of 18–55 years old. NAFLD patients were diagnosed with abdominal ultrasonography and liver enzyme tests. Total requirement data were collected around medical history, anthropometry, physical activity and dietary intake. Afterwards, a trained dietitian and a face-to-face interview were used to gather a valid and reliable Food Frequency Questionnaire (FFQ). The participants reported the frequency and amount of consumption of each food item. Finally, all food items were converted to a gram per day and used for the final analysis.

The MED diet for the first time was expressed in 1995 and revised in 2003. In the revised and final model, Trichopoulou *et al.* considered nine components for the MED diet: vegetables, nuts and fruits, cereals, legumes, fish, monounsaturated fatty acid/polyunsaturated fatty acid ratio (MUFA/SAFA ratio), low-fat dairy, meats and alcohol intake. To calculate the MED diet score, all components were obtained based on a gram per day. These values were then converted as per 1000 kcal energy intake, and sex-specific median values were used for the calculation scoring components based on the Trichopoulou method. Due to refusing to answer the questions related to alcohol consumption, this component was excluded, and finally, the MED diet score was calculated based on eight components.

We excluded patients with the following characteristics: over- or under-reporting for energy intake (*n* 20), subjects providing incomplete information (*n* 10) and subjects who had three or more components of metabolic syndrome (*n* 20). Finally, 197 subjects were involved in the analysis. Anthropometric data (height, weight and waist circumference (WC)) were measured by a trained person and a standard method. Fasting blood samples were collected after 12 h of fasting. The samples were immediately centrifuged and frozen at −80 °C. Finally, at the end of sampling, the concentration of blood sugar, total cholesterol (TC), LDL, HDL, triacylglycerols (TAG), aspartate aminotransferase (AST) and alanine aminotransferase (ALT) were determined using a standard method.

The liver steatosis was estimated by evaluating the image brightness of the echo pattern. In this regard, patients with a total liver weight of lower than 33 % were categorised as controls. Individuals were excluded at baseline in the case of (1) using drugs inducing hepatotoxicity (tamoxifen, steroids and amiodarone); (2) having cardiovascular diseases (coronary artery disease or congestive heart disease), diabetes type 1, chronic B or C hepatitis virus infections, cancer, Wilson's disease, haemochromatosis, biliary diseases or cirrhosis and another liver disease; and (3) having a history of being on a special diet. Laboratory data were collected from controls and NAFLD patients after more than 12 h of fasting in enrolment. To determine the serum concentrations of the hepatic enzymes as well as the glucose and lipid profiles, concentrations of liver enzymes, including ALT, AST and γ-glutamyltransferase, fasting blood glucose and lipid profiles, such as LDL-cholesterol, HDL-cholesterol, TC and TAG were determined.

After signing the informed consent forms, all participants underwent an abdominal ultrasound by the same radiologist using the same device. The liver steatosis was estimated by evaluating the image brightness of the echo pattern. Abdominal ultrasound cannot detect hepatic fat deposition in the case that it is less than 33 % of the total liver weight. In this regard, patients with a total liver weight of lower than 33 % were categorised as controls.

### Ethical consideration

This study was conducted according to the guidelines laid down in the Declaration of Helsinki, and all procedures involving human subjects were approved by the Human Research Ethics Committees of Shahid Sadoughi University of Yazd Medical Sciences (No. IR.SSU.SPH.REC.1399.088). Written informed consent was obtained from all subjects after explaining the process of the present study.

### Statistical analysis

SPSS software version 20 was used to perform all statistical analyses. Normality variables were examined with a histogram curve. To study continuous variables with normal and abnormal distribution, the one-way ANOVA and the Kruskal–Wallis test were used, respectively. Multivariate logistic regression models were also used to assess the relationship between the MED diet score and NAFLD risk. The MED diet tertiles were composed, and the first tertile was considered as a reference group.

## Results

In this study, the prevalence of NAFLD in people with low, moderate and high adherence to the MED diet was 33, 13⋅1 and 4⋅6 %, respectively. The means of age and the MED diet scores in the NAFLD group compared to the healthy group were 44⋅07 ± 10⋅36 *v*. 43⋅31 ± 12⋅15 years and 4⋅05 ± 1⋅27 *v*. 4⋅16 ± 1⋅38, respectively. The NAFLD group compared to the healthy group had higher BMI (31⋅85 ± 4⋅12 *v*. 25⋅40 ± 3⋅94, *P* < 0⋅001), WC (102⋅50 ± 12⋅63 *v*. 93⋅30 ± 9⋅78, *P* < 0⋅001), TAG (186⋅52 ± 82⋅63 *v*. 133⋅07 ± 59⋅00, *P* < 0⋅001), TC (177⋅22 ± 43⋅73 *v*. 160⋅43 ± 40⋅86, *P* < 0⋅006), AST (28⋅60 ± 14⋅90 *v*. 17⋅60 ± 5⋅52, *P* < 0⋅001) and ALT (39⋅12 ± 12⋅26 *v*. 19⋅76 ± 8⋅62, *P* < 0⋅001) and lower HDL (42⋅08 ± 9⋅35 *v*. 46⋅73 ± 18⋅07, *P* < 0⋅023) (data cannot be shown).

The participants in the top tertile of the MED diet score have the lower significant hip circumference (HC). There was no significant association between increasing the MED diet score tertiles and BMI, WC, FBS, lipid profile, AST and ALT (Table 1). A correlation between the MED diet score and liver enzymes was not observed in the Pearson test (AST (*r* 0⋅051, *P* 0⋅48), ALT (*r* 0⋅042,*P* 0⋅056)).

**Table 1.** Demographic, anthropometric and biochemical characteristics of the study participants according to MDS tertiles

	*T*1	*T*2	*T*3	*P-*value^a^
	≤4	≤5	≥6	
MDS (0–9)^b^	3⋅26 ± 0⋅83	5⋅00 ± 0⋅00	6⋅24 ± 0⋅51	0⋅001
Age, year	43⋅54 ± 12⋅00	45⋅57 ± 10⋅68	41⋅41 ± 10⋅91	0⋅31
BMI, kg/m^2^	29⋅35 ± 7⋅60	28⋅47 ± 5⋅51	26⋅13 ± 3⋅75	0⋅07
WC, cm	99⋅24 ± 13⋅86	97⋅80 ± 9⋅84	93⋅11 ± 7⋅45	0⋅06
HC, cm	108⋅02 ± 10⋅18	106⋅93 ± 10⋅92	102⋅30 ± 6⋅26	0⋅02
FBS, mg/dl	98 (92–118)	100 (93–112)	101 (93–119)	0⋅67
TAG, mg/dl	163⋅32 ± 84⋅23	16⋅20 ± 67⋅35	146⋅33 ± 53⋅90	0⋅57
TC, mg/dl	168⋅77 ± 41⋅31	174⋅95 ± 42⋅07	163⋅29 ± 49⋅52	0⋅52
LDL, mg/dl	105⋅93 ± 32⋅61	103⋅73 ± 31⋅03	113⋅22 ± 46⋅35	0⋅51
HDL, mg/dl	42 (38–47)	42 (35–48)	43 (36–50)	0⋅98
AST, IU/l	24⋅04 ± 11⋅54	23⋅25 ± 12⋅43	22⋅62 ± 12⋅20	0⋅84
ALT, IU/l	29⋅67 ± 22⋅38	30⋅52 ± 19⋅87	29⋅30 ± 23⋅40	0⋅95
				
Male, *n* (%)	52 (26⋅4)	23 (11⋅7)	10 (5⋅1)	0⋅35
Diabetes, *n* (%)	35 (17⋅8)	14 (7⋅1)	7 (3⋅6)	0⋅81
Physical activity, *n* (%)	79 (40⋅1)	27 (13⋅7)	21 (10⋅72)	0⋅55
Prevalence of NAFLD, *n* (%)	65 (33⋅1)	26 (13⋅2)	9 (4⋅6)	0⋅06

MDS, Mediterranean dietary score; BMI, body mass index; WC, waist circumference; HC, hip circumference; FBS, fasting blood sugar; TAG, triacylglycerols; TC, total cholesterol; LDL, low-density lipoprotein; HDL, high-density lipoprotein; ALT, alanine aminotransferase; AST, aspartate aminotransferase.

a ANOVA or Kruskal–Wallis test for a continuous variable and *χ*^2^ test for a categorical variable.

b Per 1000 kcal energy intake.

The distribution of the MED diet components and macronutrient intake across the MED diet score tertiles are exhibited in Table 2. The increasing intake of the MED diet was significantly related to the increment intake of vegetables, nuts and fruits, cereals, legumes, fish and MUFA/SAFA ratio, albeit the consumption of total energy, low-fat dairy and meats intake was decreased. The relationship between the MED diet score and the risk of NAFLD is presented in Table 3. In the crude model, the individual in the highest MED diet tertile compared to the lowest tertile and the risk of NAFLD to 60 % (OR: 0⋅40, 95 % CI: 0⋅17–0⋅95) were decreased. In model 1, after controlling for sex, age, diabetes, physical activity and supplement intake, the relationship became stronger (OR: 0⋅36, 95 % CI: 0⋅15–0⋅89). However, in model 2, after adding BMI, WC and HC to model 1, this relationship disappeared (OR: 0⋅70, 95 % CI: 0⋅25–1⋅97). Crude and adjusted OR for NAFLD according to MUFA/SAFA ratio tertiles were reported in Table 4. Individuals in the highest MUFA/SAFA ratio tertiles have lower odds for NAFLD in crude and after adjusteing for age, sex, diabetes, physical activity and supplement intakead.

**Table 2.** Distribution of the MED diet components and macronutrient intake across the MED diet score tertiles

	*T*1	*T*2	*T*3	*P* -value^a^
	*N* 123	*N* 45	*N* 29	
Energy, kcal/d	2352⋅86 ± 680⋅80	1924⋅01 ± 666⋅40	1702⋅04 ± 486⋅72	0⋅001
Vegetables, g/1000 kcal	138⋅04 ± 83⋅72	165⋅30 ± 80⋅06	181⋅76 ± 65⋅60	0⋅001
Cereals, g/1000 kcal	132⋅87 ± 55⋅30	139⋅08 ± 51⋅73	163⋅15 ± 60⋅35	0⋅031
Legumes, g/1000 kcal	12⋅86 ± 8⋅46	18⋅34 ± 10⋅85	21⋅30 ± 9⋅40	0⋅001
Fruits and nuts, g/1000 kcal	309⋅39 ± 155⋅71	362⋅65 ± 152⋅14	382⋅42 ± 148⋅21	0⋅013
Fish, g/1000 kcal	5⋅93 ± 10⋅70	5⋅79 ± 3⋅48	7⋅21 ± 7⋅77	0⋅021
Low-fat dairy, g/1000 kcal	180 (92–298)	147 (96–261)	142 (93–277)	0⋅004
Meats, g/1000 kcal	62⋅80 ± 31⋅70	54⋅25 ± 23⋅70	40⋅54 ± 18⋅60	0⋅001
MUFA/SAFA ratio, g/1000 kcal	0⋅47 ± 0⋅20	0⋅59 ± 0⋅24	0⋅63 ± 0⋅17	0⋅001
Carbohydrate, g/d	329⋅86 ± 112⋅36	281⋅87 ± 114⋅20	262⋅21 ± 78⋅10	0⋅001
Protein, g/d	100⋅91 ± 37⋅09	80⋅48 ± 32⋅24	65⋅38 ± 22⋅12	0⋅001
Total fat, g/d	82⋅26 ± 28⋅39	62⋅00 ± 19⋅90	52⋅41 ± 15⋅34	0⋅001
Tea and coffee, g/d	624⋅89 ± 187⋅17	285⋅84 ± 215⋅44	240⋅52 ± 192⋅87	0⋅001

a ANOVA or Kruskal–Wallis test for a continuous variable and *χ*^2^ test for a categorical variable.

**Table 3.** Crude and adjusted OR for NAFLD according to MED score tertiles

MED	*T*1	*T*2	*T*3	*P* for trend	*P*-value
	≤4	5	≥6		
Crude	1⋅00	1⋅22 (0⋅61–2⋅43)	0⋅40 (0⋅17–0⋅95)	0⋅02	0⋅038
Model 1	1⋅00	1⋅20 (0⋅60–2⋅47)	0⋅36 (0⋅15–0⋅89)	0⋅04	0⋅027
Model 2	1⋅00	1⋅31 (0⋅55–3⋅15)	0⋅70 (0⋅25–1⋅97)	0⋅13	0⋅58
Model 3	1⋅00	1⋅60 (0⋅63–3⋅05)	0⋅88 (0⋅30–2⋅65)	0⋅22	0⋅81
Model 4	1⋅00	1⋅52 (0⋅73–3⋅14)	0⋅54 (0⋅22–1⋅35)	012	0⋅71

Model 1: adjusted for age, sex, diabetes, physical activity and supplement intake.

Model 2: Model 1, additional BMI, WC and HC.

Model 3: Model 2, additional total energy intake.

Model 4: Adjusted for total energy intake.

**Table 4.** Crude and adjusted OR for NAFLD according to MUFA/SAFA ratio tertiles

MUFA/SAFA ratio	*T*1	*T*2	*T*3	*P* for trend	*P*-value
	≤0⋅42	0⋅43–0⋅56	≥0⋅57		
Crude	1⋅00	1⋅33 (0⋅66–2⋅67)	0⋅47 (0⋅24–0⋅95)	0⋅013	0⋅04
Model 1	1⋅00	1⋅30 (0⋅63–2⋅62)	0⋅42 (0⋅20–0⋅87)	0⋅03	0⋅007
Model 2	1⋅00	1⋅85 (0⋅78–4⋅35)	0⋅71 (0⋅28–1⋅76)	0⋅14	0⋅09
Model 3	1⋅00	2⋅50 (0⋅88–7⋅11)	1⋅32 (0⋅30–6⋅10)	0⋅11	0⋅70
Model 4	1⋅00	1⋅90 (0⋅81–4⋅44)	0⋅92 (0⋅30–2⋅92)	0⋅15	0⋅09

Model 1: Adjusted for age, sex, diabetes, physical activity and supplement intake.

Model 2: Model 1, additional BMI, WC and HC.

Model 3: Model 2, additional total energy intake.

Model 4: Adjusted for total energy intake.

## Discussion

The present study revealed a reverse association between high adherence to MED diet and NAFLD odds, even though this relationship was disappeared after further adjustment for the anthropometric variable. Similar to some other studies, results, nested and matched case-control^(16)^ and cross-sectional^(17)^, reported that adherence to the MED diet in any crude and adjusted models was not associated with the risk of NAFLD. In addition, Kontogiani *et al.*, in a cross-sectional study, did not observe a difference between adherence to the MED diet in NAFLD compared with control groups. However, this dietary pattern had an inverse association with the severity of NAFLD^(18)^. However, some studies inconsistent with the present study, including Bullon-Vela *et al.*, claimed that greater adherence to the MED diet scores was inversely associated with hepatic steatosis index in a cross-sectional study of PREDIMED-PLUS, which is a large multi-centre clinical trial in the multivariate linear regression^(19)^. In a cross-sectional study, Barrata *et al.* reported that the MED diet score obtained based on a 9-item semi-quantitative questionnaire was negatively associated with the risk of NAFLD in the adjusted model^(20)^. Besides, Aller *et al.*, in a cross-sectional study, indicated that higher adherence to the MED diet score was associated with a lower chance of a high grade of steatosis^(21)^. A recent meta-analysis also confirmed that Mediterranean dietary patterns reduced the risk of this disease by 23%^(22)^.

The present study did not explore a significant relation between the MED diet score with ALT and AST. These results were similar to some other studies, including Della Corte *et al.*, in which a relation between good, moderate and low adherence to KIDMED (the Mediterranean Diet Quality Index for children and adolescents) and the rise of ALT and AST were not observed^(23)^. Also, Tzima *et al.* illustrated that adherence to the MED diet in the metabolic syndrome patients was not relevant to reducing liver enzyme levels^(24)^.

The mean adherence to the MED diet score in the present study was 4⋅02 ± 1⋅7. It was similar to some other investigations^(16)^. It seems that the difference in the amount of sex-specific median intake of components in the MED diet could be related to the beneficial effects of this dietary pattern on NAFLD prevention. For example, males and females in the Trichopoulou study were compared to males and females in the present study who consumed more vegetables (median intake in male: 550 *v*. 278 g/d, in female: 500 *v*. 280 g/d), MUFA/SAFA ratio (median intake in male: 1⋅7 *v*. 1, in female: 1⋅7 *v*. 0⋅9) and fish (median intake in male: 24 *v*. 10 g/d, in female: 19 *v*. 6 g/d).

Several mechanisms may be related to the beneficial effects of the MED diet on NAFLD because of the low content of simple sugars and fructose. The high content of soluble and insoluble fibres is associated with a decrease in serum TAG and blood glucose^(25)^. This dietary pattern also has plenty of fruit, vegetables, olive oil and antioxidant compounds (twenty-eight), which may be particularly useful in NAFLD patients^(26)^.

The protective effect of the MED diet on the fatty liver was disappeared after further adjustment for BMI and WC and HC as central obesity, which shows that the MED Diet can play a role in improving fatty liver through weight modification, lipid profile and inflammation markers^(27,28)^. Several mechanisms have been proposed to explain the association between obesity and fatty liver, including changes in cytokines, inflammation factors, insulin resistance, dyslipidemia^(29)^ and regulation of the expression of some of the genes (PPAR-γ and others)^(30)^. It is suggested to conduct future studies with more participants and BMI-matched case-control studies to determine the differences between groups more specifically.

Some strengths of the present study include the following issues: the questionnaires were completed by a trained interviewer blinded to the participants’ categorisation in the case or control group, which minimised the reporting error. Also, we used newly diagnosed individuals with NAFLD (incident case) as the case group. Various confounders associated with fatty liver and also dietary patterns were adjusted. The limitation of the present study includes the possibility of recall bias due to the nature of observation. However, in order to decrease this bias, a valid and reliable FFQ was used. Due to the case-control design of the study, determining a clear causal relationship was impossible between dietary pattern adherence and NAFLD. Since the case group members included newly diagnosed NAFLD patients, the probability of changing their dietary patterns was low after the disease diagnosis.
